# Denial of Access to Individuals Seeking Inpatient Care: Disposition Determinants and 12 Month Outcomes

**DOI:** 10.19080/jfsci.2017.02.555592

**Published:** 2017-03-28

**Authors:** Steven P Segal, Perri Franskoviak

**Affiliations:** 1 Mental Health and Social Welfare Research Group, University of California, USA; 2 Holy Names University, USA

**Keywords:** Civil Commitment, Access to Inpatient Care, Psychiatric Emergency Services, PES, General Hospital Psychiatry, Managed Care, Dangerousness, Psychiatric Hospitalization

## Abstract

**Objective:**

This study considers the denial of access to inpatient care to those seeking hospitalization following psychiatric emergency service (PES) evaluation. It evaluates how civil commitment criteria, functional status, institutional constraints, social bias, and procedural justice indicators are likely to impact denial of care decisions, and considers 12 month outcomes.

**Methods:**

PES evaluations of 583 patients in 9 California county general hospitals were examined via logit modeling to determine those factors contributing to the decision to deny access to inpatient care. Differences in the importance of influences on the decision making process and outcomes at 12 months are examined in two contrasts: first, admitted and released patients seeking care, then, the latter group versus all other patients. Outcome measures include numbers of deaths, violent crimes, and involuntary readmissions to the PES.

**Results:**

Of the patients evaluated, 8.4 % were denied access to inpatient care despite their avowed wish to be hospitalized. When compared to admitted patients seeking hospitalization or to all other patients, analyses show that clinicians relied on civil commitment admission criteria and the availability of a less restrictive alternative to the hospital in making decisions on patient retention. When compared with all other patients, the probability of unwanted release was greater for individuals evaluated in difficult circumstances, for those without insurance, and for those with higher functional status. Fewer deaths were observed in the group denied admission, though no other significant outcome differences were observed.

**Conclusion:**

Dangerousness and mental disorder in the absence of a less restrictive alternative to hospitalization, along with an overall assessment of the patient’s functional status, are effectively employed as triage criteria in determining who is denied access to inpatient care following PES evaluation. While some higher functioning individuals are subjected to a variant standard of access to inpatient care because of a lack of insurance, and endure the misfortune of being evaluated under difficult clinical circumstances, outcomes seem contingent on clinicians’ ability to distinguish between groups on the aforementioned triage criteria.

## Introduction

There is a need for a better understanding of clinical decision making in the psychiatric emergency service (PES) civil commitment evaluation disposition process. The civil commitment criteria for involuntary detention, danger to self or others due to a mental disorder, appear to have become a de facto rationing or triaging mechanism for an inpatient care disposition following the PES evaluation. Yet, all admissions to hospital are not necessarily unwanted. There are those individuals who come to the PES seeking inpatient care. Regardless of the benefits associated with hospitalization, such an action is an act of some despair. In the past, the hospital was a bed of last resort to all comers. This is no longer true today. In turning away those who would seek inpatient care, there is a potential deprivation of the benefit of treatment to those who are released. This would be especially egregious if the denial of hospitalization results from factors other than failure to meet the de facto criterion for the receipt of such care, or results in adverse outcomes potentially preventable via hospitalization.

For instance, in 1997, Andrew Goldstein A….walked into the Creedmoor [State Hospital] lobby, asking to be admitted. “I want to be hospitalized”, he said…but in a cost-cutting drive…he…was released [1]. Mr. Goldstein subsequently pushed Kendra Webdale off a platform in front of a New York subway train. His case laid the foundation for the broadening of New York’s commitment laws. Was it an expanded commitment law that was needed to address Mr. Goldstein’s situation or, perhaps, more attention to the nature and quality of the civil commitment evaluation? Denial of care requests for such patients can be catastrophic for the community. While there is no right to treatment guaranteed in the community, fairness and prudence would have us consider in greater detail those factors that influence the decision to deny inpatient treatment, especially to that subset of individuals seeking hospitalization who are often thought to be sounding an alarm, or simply crying out for help to prevent their impending decompensation.

### The PES evaluation as a civil commitment evaluation

The PES in general hospitals is where most civil commitment evaluations are completed and where significant numbers of psychiatric inpatient stays are approved. As such, it is often the first stage in the mental health system where the patient’s dangerousness and mental disturbance is assessed by trained and experienced professionals under psychiatric supervision, and where the decision is made to release or retain the patient. PES clinicians, in California, may retain the patient based upon the facts of the case (exclusive of hearsay evidence) for 72 hours for observation and treatment with probable cause evidence indicating that the patient meets the standard of danger to self or others, or gravely disabled due to a mental disorder, in the absence of a less restrictive alternative [2]. Usually patients are committed to the hospital’s own inpatient facility, or to a private hospital inpatient facility in the area. The patient is less frequently referred to a state facility.

This paper considers how the PES evaluation, conducted by the most specifically trained and experienced personnel in the system (i.e., in their ability to consider the dynamics of mental illness and dangerous behavior), operates to protect the rights of individuals and the good of the community. PES evaluations in general hospitals may be considered the result of a complex negotiation whose character is not always evident, and where the factors involved in disposition decisions are not well understood. The PES evaluation, for example, is no longer a simply described coercive event where people are forced to sign voluntary admission orders when they do not wish to be admitted to a hospital [3]. In fact, many individuals come to the general hospital PES seeking “involuntary” care because that is the only way they can get care. For this reason, and because inpatient care represents the most restrictive form of treatment, it is necessary to insure that the denial of inpatient care to those seeking it under such difficult circumstances is an action taken on behalf of the individual and the community, without the influence of inappropriate or confounding issues unrelated to the primary admission criteria. In making such a determination, we consider five sets of factors with the potential to influence the outcomes of the PES disposition decision process: patient standing on the civil commitment criteria, patient functional status, institutional constraints, procedural justice characteristics, and social biases.

### The PES evaluation and civil commitment criteria

Because of the legal and clinical importance of an involuntary commitment decision, gathering the information necessary to make a determination of the patient’s status on the civil commitment criteria and making a decision regarding involuntary admission to care is the core content of the PES evaluation. Mental disorder and dangerousness represent the prevailing involuntary admission criteria in civil commitment statutes in most? states. In California, these criteria can only be used in the absence of “a less restrictive alternative to hospitalization” [2]. On the other hand, “treatability” and “ability to benefit from hospitalization” represent measurable aspects of a “need for treatment criteria” approach proposed by advocates of the APA Model Law [4,5]. Moreover, mental disorder and dangerousness may also be viewed as indicators of the patient’s problematic interaction with society, while treatability and the ability to benefit from hospitalization, as well as the availability of a less restrictive alternative to inpatient care, can be considered to be measures of the patient’s fit with the mental health system. While these latter criteria are the basis for admission in several states, and their use in the evaluation is advocated by some as an appropriate expansion of clinical discretion, in a state with a “dangerousness criterion” statute their use may be viewed by others as a violation of due process.

If the civil commitment criterion prevailing in a state becomes the de facto triage mechanism used to dispense inpatient care, what are the consequences (societal, individual, and clinical) of relying on a dangerousness criterion that would retain those individuals whose problems with society may be greatest, as opposed to those whose needs the mental health system may more easily meet, or whose behavior may be more easily controlled? Does clinician discretion in admissions decisions become bound by the civil commitment criteria? Are those individuals who are denied admission when they want it patients who fit the “treatability” and “ability to benefit” standards, but whose problems with society may deem them as less dangerous, and therefore less eligible for hospitalization? While we cannot compare the two civil commitment standards, we will look at the basis for releasing patients who are seeking hospitalization. We do so by assessing the clinician’s reliance on the civil commitment criteria as a de facto triage standard. We also evaluate the outcomes of the “dangerousness-based” disposition decision process to determine whether those individuals who demonstrate greater fit with the mental health treatment system are in fact deselected from the inpatient care they are seeking, and evidence adverse consequences within 12 months following their evaluation, i.e., death, involuntary admission to a hospital, and post-admission involvement in violent crime.

### Influences on PES disposition decisions

Considering that most clinicians view themselves as acting in the patient’s best interest, we examined whether clinician discretion in admissions decisions becomes bound by the civil commitment criteria, or whether clinicians also take into account the patient’s global functioning and/or an alternative symptom severity based-definition of mental disorder in considering their decision to release a patient seeking inpatient care. PES disposition decisions may also be influenced by institutional constraints such as those attributed to the increasing use of managed care strategies to limit inpatient admissions. These constraints include increasing workloads, treatment decisions based on the patients’ insurance coverage, and the use of difficult, inadequate and unaccommodating work environments-spaces that add greater burden to the practitioner, which may lead to inappropriate release decisions. Procedural justice issues, salient in the 1960s, continue to be of concern in contemporary PES settings, where inappropriate advocacy, institutional processing, and inadequate patient participation in the evaluation process have a tendency to occur [6]. Finally, the impacted race and gender social bias on decision-making in the PES setting may lead to inappropriate disposition decisions [7,8].

This paper will consider the relative importance of constraints, biases, and procedural justice issues in predicting denial of access to inpatient care following evaluation in the PES. Particular emphasis will be placed on determining the relative importance of an individual’s standing on the criteria for civil commitment as the standard for deciding who needs care following an evaluation. Since these criteria are meant to provide a framework for justifying the limited circumstances under which inpatient care is offered, ideally we should expect that no constraints, biases, or procedural justice issues should influence the decision to deny such care. Such actions should almost entirely be determined by the patient’s standing on the admission criteria. Previous investigations have demonstrated the primacy of these criteria in admission decisions [9–11]. However, this study moves beyond previous investigations by considering the relationship of admission criteria to the decision to deny inpatient care to those who actually want it. We further specify the types of constraints, biases, or procedural justice issues which may influence the denial of care outside of the primary clinical criteria, and consider the relationship between denial of access to inpatient care and adverse outcomes at 12 months following the index PES evaluation.

## Methods

### Sample and procedures

Of 711 attempted observations of patients who had visited one of nine PESs during a two year period, 683 participated in the study. The refusal rate was 3.9% (n=28), meaning that these cases were not included in the analysis due to their own preference or the preference of their PES clinician. Following the first 100 observations (which focused primarily on the dangerousness assessment), the study protocol was expanded to obtain the information necessary to answer the questions posed herein. This study, therefore, considers the evaluations of the 583 patients who were empanelled under the expanded protocol guidelines. PES observations were obtained from seven San Francisco Bay Area, one Los Angeles, and one California Central Valley site. To insure narrow confidence intervals on validity estimates in dangerousness assessments, the primary purpose of the original study, a minimum of 50 observations were obtained from each PES. Sites outside the Bay Area were selected to expand the generalizability of the findings. Including Los Angeles and Fresno gave us the opportunity to look at differences between PES practices in areas that functioned under the same legal and clinical criteria, yet differed significantly in terms of socio-cultural make-up. Informed consent for human investigation was obtained from all study participants. Assessments were observed in an apparently random manner. Subjects were chosen consecutively on entry to the PESs and observations were completed around the clock seven days a week.

As soon as one case was completed the next one was assessed. No case was passed over for any reason other than the case’s refusal to participate. The observer accompanied the patient and the PES clinician throughout the course of the assessment, witnessing all interactions including telephone contacts, and was privy to all information available to the clinician. The observer recorded the entire assessment process until a disposition decision was reached by the PES clinician. In addition to information about the patient that had been gathered by the clinician, the observer coded her/his own impressions about the patient and several aspects of the PES clinician’s treatment of the patient. Information was ascertained and recorded on structured scales, as well as recorded in the form of process notes. Observer ratings were not available to the clinician. Acceptable inter-rater reliability between observers, on key study instruments, was established before the independent observations were initiated. Human subjects procedures were reviewed and approved by 11 committees. Follow-up information is based on public health, criminal justice, and medical record review covering the 12-twelve months following the evaluation.

### Measures

#### A. Denial of inpatient care following PES evaluation:

In order to define this subgroup of patients, we took into account the expressed wishes of the patient during their PES evaluation as to whether or not they wanted hospitalization. Patients seeking hospitalization who were released against their wishes were considered to be those who were denied access to inpatient care.

- Admission criteria/severity of the patient`s condition: Four affirmative admission criteria that are consistent with current and proposed legal requirements are analyzed as indicators of the severity of the patient’s condition:
  - clinician assigned DSM III diagnosis of a Psychotic Disorder,
  - the patient’s likelihood of causing harm to self, harm to others, or being gravely disabled at the time of the PES evaluation, the TRIAD Dangerousness Scale Score (Three Ratings of Inpatient Admissibility) [12] [range 1–11, higher scores indicate increased dangerousness],
  - whether or not the psychiatric disorder was viewed as treatable by the clinician, the Treatability Scale Score [12,13] [range 0–1, higher scores indicate greater Treatability], and
  - The patient’s ability to benefit from hospitalization, Benefit from Hospitalization Scale Score [12,13] [range 0–1 higher scores indicate greater likelihood of benefitting from hospitalization].

In addition, we included the presence of a less restrictive alternative placement as an obviating factor, whether or not it is wanted by the patient. The presence of such an alternative (defined as any supervised residential arrangement, including placement with a willing and responsible relative, crisis housing, nursing homes, and foster family care) was measured as a 1/0 variable with 80% inter-evaluator agreement.

#### B. Functional status:

Mental disorder and dangerousness represent the prevailing admission criteria in California, and should be the sole object of the assessment for involuntary hospitalization. Yet, patients seeking inpatient care may be considered voluntary or at least potentially voluntary, and therefore, their overall functional status becomes an issue. Patient’s functional status at the outset of the evaluation was measured by clinician ratings on Spitzer and Endicott’s Global Assessment Scale (GAS) [14]. We further considered a symptom-based measure of mental illness, derived from the Massachusetts law, the Indicators of Mental Disorder Scale (IMDS) [11,15,16], as an alternate clinical indicator of a patient’s functional status, which is not used as a legal criterion defining mental illness under California Law (LPS). Finally, in order to be able to understand the meaning of the PES interaction, the patient’s credibility was rated by observers on a ten-point scale.

#### C. Procedural justice:

Procedural justice indicators tell us that the evaluation process has been carried out in a manner that would lead an impartial observer to conclude that a serious and unbiased effort was taken to determine the patient’s status on the legal admission criteria. Three indicators of procedural justice were used.

##### I. Involuntary legal status at PES entry:

While not a direct measure of procedural justice, a very strong relationship between this variable and involuntary detention is hypothesized by deviance theorists. Deviance theorists [6] argue that civil commitment proceedings are lacking in procedural justice, whereby persons arriving at the PES are routinely processed and retained or released consistent with prior statuses or labels. Under such circumstances prediction of the patient’s disposition would be highly associated with legal status at entry to the PES. This assumption is testable by the inclusion of this indicator in our PES decision evaluation model as a predictor of disposition.

##### II. The art of care scale:

The primary concern in assessing procedural justice is insuring that the process is conducted fairly [17–19]. This can only be achieved when the patient has been given the chance to fully participate in the evaluation to the maximum extent possible [3,19–21]. The Art of Care Scale, though designed to measure one aspect of quality of care [22], measures the extent and character of such participation. It includes the average of four items (scored 1 if present, 0 of absent) which address the clinician’s attempt to engage in a collaborative interaction, elicit information from the patient, include the patient in planning appropriate to their functioning, and attend to the patient’s feelings with empathy. Inter-rater agreement in coding the items from process notes averaged .75 and the internal consistency, Alpha = .69.

##### III. Advocacy for and/or against hospitalization:

An additional concern in civil commitment evaluations has been the inappropriate influence of advocates in the PES evaluation process. Given the patient’s possible failure to exercise a free choice in entering a psychiatric hospital, undue influence on the part of others whose preference may dominate may lead to inappropriate disposition decisions [23,24]. The influence and role of advocates on disposition decisions will be evaluated.

#### D. Institutional constraints:

Factors that might be considered institutional constraints on the clinician’s disposition decision include:

- excessive clinician’s workload (measured by a four-item factor score including patient-staff ratio in the PES [Factor weight = .257], the clinician’s patient load [Factor weight = .683], and the total number of inpatient beds [Factor weight = −.132] and out of hospital beds [Factor weight = −.168] available at the time of the evaluation);
- difficult circumstances in which the evaluation was completed (measured as a 1/0 rating based upon the observer’s conclusion that the patient was assessed in a context including conditions of: relentless noise, limited space, limited phone access, visual distractions, and/or other negative characterizations); and,
- Absence of insurance coverage (measured as a 1/0 rating, where Medicaid and Medicare were included as a form of insurance).

#### E. Social bias indicators

Social bias indicators that might prejudice a clinician toward implementing a coercive, or undesirable, disposition include demographic characteristics which have conventional association with discrimination-i.e., patient’s gender (coded 0=male and 1= female) and ethnic minority status (coded 1 = African American and 0 = other).

#### F. Other context controls

These included: time of evaluation (9AM-5PM vs. other), hospital in which evaluation was completed (nine 0/1 dummy variables), technical quality of care received [2], the experience of the evaluator, and whether the disposition was voluntary or involuntary.

### Analyses

In looking at the situation of released patients who were seeking hospitalization, we first compare them to all other patients. We then consider the disposition issue by comparing the experience of those seeking hospitalization who were released to two contrasting groups: all other patients entering the PES, and those patients entering the PES who were seeking hospitalization and were retained. This comparison may offer a closer look at the factors involved in the clinician’s decision to deny access to care. Univariate/bivariate. The demographic characteristics of the sample will be reported along with bivariate analyses on all variables distinguishing comparison groups. Also considered are the 12 month outcomes of those denied hospitalization in comparison to the two contrast groups. Bivariate relationships are evaluated using t tests for differences in means and Chi-square analyses for categorical comparisons.

#### I. Multivariate:

We first evaluate our theoretical model with a two stage logit regression, focusing on those factors most likely to distinguish individuals in the denied access group from all other patients. The model’s first stage examines the relative importance and significance of admission criteria, functional status, procedural justice indicators, institutional constraints, and social biases. In the second stage a set of control factors are entered. This model was run three times with a different set of controls entered with each iteration. The first set includes quality of care issues measured by clinicians’ experience and the Gustofson’s Technical Quality of Care Scale [22]. The next set of controls entered in the second stage of the regression involves methods variables (time of entry into the PES, time of retention/ release decision [both measured as a 0/1 variables of 9AM −5PM versus other], and hospital in which the decision was made [eight of nine possible 1/0 dummy variables]). Finally, the model is run with a 1/0 dummy variable indicating whether or not the retention was voluntary or involuntary.

In a second phase of our multivariate analysis, we work only with patients seeking hospitalization. We use the variables specified in our theoretical model in a stepwise logistic regression predicting patient disposition only among those seeking hospitalization. (IMDS scores are not used in the multivariate models given their more limited availability and therefore their adverse effect on sample size.)

## Results

### Characteristics of patients and clinicians

The modal patient was white (66%), male (56%), age 27, and was English-speaking (94.7%). The mean age was 35.6 years. Only 2.4% spoke no English. Minorities were well represented in the sample, which included 18.9% Black, 10.8% Spanish Surname, 1.5% Asian and 2.7% other minorities. The mean number of years since the patient’s first psychiatric hospitalization was 8.19 (sd +8.93), the mean number of prior hospitalizations was 3.94 (sd + 7.92), and the mean number of previous visits to the PES in which they were evaluated was 4.02 (sd + 8.98). Before coming to the PES for the index evaluation, the number of patients with a criminal record involving a felony was 247 (36.2% of the sample), the number convicted of a felony was 177 (25.9% of the sample), the number convicted of a violent felony was 37 (5.4 % of the sample), and the number convicted of a sex-related felony was 17 (2.5% of the sample). In the year following their PES index evaluation, the number of patients with a criminal record involving a felony was 175 (25.6 % of the sample), the number convicted of a felony was 105 (15.4 % of the sample), the number convicted of a violent felony was 17 (2.5 % of the sample), and the number convicted of a sex related felony was 11 (1.6 % of the sample). PES clinicians were primarily psychiatrists or other physicians (50%), but they also included registered nurses (7.7%), master’s-level psychologists (10.6%) and social workers (7.0%), licensed psychiatric technicians (9.1%), other trainees (2.4%), Ph.D. psychologists (2.1%), and persons with other credentials (10.5%). Most non-psychiatrists had a psychiatrist available for consultation. Evaluators were 85.6% white, with 6.7% Spanish surname, 5.1% black, 2.2% Asian, and .2% other. Minority clinicians saw about 50% more than their proportionate share of minority cases, yet an ethnic match was not available for every client. The evaluators had an average of 5.5 years of experience in the psychiatric emergency room. Patients preferring hospitalization did not differ from all other patients on any of the aforementioned characteristics.

#### a. Admission criteria:

The average TRIAD Dangerous Score was 3.2, making the average patient severe enough to be civilly committed on any one of the three dangerous criteria: Danger to Self, Danger to Others, or Gravely Disabled. Although nearly 95% were considered to have some mental disorder, only 61.7% were found by clinicians to have a psychotic disorder. A third of the patients were above the mean on the Treatability Scale, and 25% of the sample were viewed as able to benefit from hospitalization. A less restrictive alternative to hospitalization was available for 54.5% of the patients. Patients preferring to be hospitalized were more likely to be viewed as treatable and able to benefit from hospitalization (t=2.01, df=436, p=.028); though they were neither more dangerous on TRIAD assessments than other patients, nor less likely to have a less restrictive alternative to hospitalization available to them.

#### b. Functional status:

Client functioning at entry was rated on the Global Assessment Scale (GAS) (Mean = 35.6, sd + 13.6; Median=35). 74% of the sample were of a severity appropriate to receive acute treatment (GAS score 40 and below [14]). These patients had functioning levels varying from “major impairment” to needing “constant èrvision.” While not differing in their functional status as measured on the GAS, patients preferring hospitalization did present a somewhat different symptom presentation on the IMDS. When compared to other patients they were more impulsive (Mean Preferred = 4.28 vs Mean Others= 3.62, df =309, t= 3.48, p=.001), evidenced greater levels of poor judgement (Mean Preferred = 3.94 vs Mean Others= 3.27, df= 116, t= 3.09, p=.002), showed more problematic behavior (Mean Preferred= 3.80 vs Mean Others= 2.61, df = 310, t = 5.48, p<.000), and demonstrated a greater level of anxiety (Mean Preferred = 3.03 vs Mean Others = 2.59, df = 310,t = 2.09, p=.037). They were also less depressed (Mean Preferred = 2.8 vs Mean Others = 3.24, df = 310, t = 2.04, p=.043). The average patient had a credibility score of Mean = 7.22 (sd + 1.75) out of ten. There was no difference between those patients preferring hospitalization and all others on their perceived credibility as rated by trained observers.

#### c. Indicators of procedural justice:

A majority of cases entered PES with an involuntary legal status (55.9%). Of the 583 cases in the study, advocates offered advice to hospitalize a client in 168 instances. Advice not to hospitalize was offered 35 times out of 203 advocate responses. Of all the advocates, 37.3% were relatives and friends, 33.7% were professionals, and 14.5% were interested community members, such as landlords who preferred hospitalization. Of those advocates who advised against hospitalization, 6.7% were relatives and friends, 5.7% were professionals, and 2.1% were community members. On the Art of Care Scale (range 0–1), our third procedural justice indicator, 231(39.6%) of the patients received the highest score, indicating that they were very much engaged in the process of the evaluation to the level they were capable of being so engaged. Those preferring hospitalization did not differ from other patients on any of the aforementioned procedural justice issues.

#### d. Institutional constraints:

More than a fourth (27.4%) of the patients had no insurance, and 11% had their evaluations completed under conditions that were considered difficult. The workload factor score is a function of evaluator caseload (averaging 2.25 patients at the time of evaluation, and ranging between one and nine patients), patient/staff ratio in the PES at the time of the evaluation (averaging .85 and ranging from .14 to 4.00), in house beds available (averaging 3.5 and ranging from 0 to 20), and beds available outside the hospital (averaging 5.96 and ranging from 0 to 44). Though primarily defined in the factor score by the weight given to caseload and secondarily to patient staff ratio, bed availability is an influence in the clinicians’ workload experience. Those preferring hospitalization did not differ from other patients on any of the aforementioned institutional constraints.

#### Bivariate analyses of disposition:

Within the total sample investigated, 36.1% (N=187) were released following their evaluation. Of the 24.3% (N=126) of the sample preferring hospitalization 38.9% (N=49) were released. Release was not significantly related to the patient’s expressed desire regarding hospitalization. Comparing those released who wanted inpatient care to all other patients. Bivariate differences showed that those preferring inpatient care who were released were less severe on two of the affirmative admission criteria: TRIAD Dangerousness scores (Mean Preferred & released = 1.63 (sd. + 1.8) v. Mean Others = 3.4 (sd +2.25); t = 3.6, df, 563 p<.000); and Psychotic Diagnosis (34.8% wanting hospitalization & released with a psychotic diagnosis v. 69.6% of other patients with a psychotic diagnosis; X2 =23.29, df=1, p<.000). The availability of a less restrictive alternative was also significantly related to release (95.8% v. 51.1%; X2 =35.79, df=1, p<.000). Patients who were released though preferring hospitalization were higher functioning than other patients as measured by GAS (Mean Preferred = 42.8 vs Mean Others 34.9; t= 4.55; df =51, p< .000). On three of the fourteen symptoms on the IMDS (Thought [form], Thought [content] and Irritability), released patients were significantly less disturbed (p<.027).

No differences were noted between groups on credibility. Bivariate differences favoring release of that wanting inpatient care were noted for two institutional constraints: difficult setting (26.7% vs 9.9%; X2 =11.53, df=1; p<.000) and lack of insurance (46.9% vs 26.3% were uninsured; X2 =11.12, df=1; p<.000). Comparing those released who wanted inpatient care to those retained who wanted such care. Bivariate differences between patients released and retained that wanted inpatient care were observed on two of the affirmative admission criteria: TRIAD Dangerousness scores (Mean Released = 1.63(sd +1.8) vs Mean Retained = 4.26 (sd+2.05); t = 7.3, df=120; p<.000); and Psychotic Diagnosis (22.2 % released with a psychotic diagnosis vs 77.8% retained with a psychotic diagnosis; X2 =18.82, df=1; p<.000). The availability of a less restrictive alternative was also significantly related to release (56.1% Released vs 43.9% Retained; X2 =30.85, df=1; p<.000). Patients released versus those retained did not differ on ability to benefit from hospitalization or on the treatability criteria. Ironically, patients who preferred hospitalization were less likely to be held with a voluntary status than other patients (X2 =7.36, df=2; p<.025). Only 8.3% (N=4) of those preferring hospitalization were held voluntarily in contrast to more than one quarter of other patients.

Patients preferring hospitalization who were released were higher functioning than retained patients preferring hospitalization as measured by GAS (Mean Preferred released = 42.8 vs. Mean Preferred retained 33.5; t= 4.63; df =82.23; p< .000). On eight of the fourteen symptoms on the IMDS (Table 1), released patients were significantly less disturbed than those retained. No differences were observed between groups on credibility. Bivariate differences between the released and retained individuals seeking hospitalization were not significant on any of the social bias or procedural justice indicators. Two institutional constraints differences were significant: evaluation under difficult circumstances (70.6% of the released vs 29.4% of the retained; X2 =6.37, df=1; p<.012) and lack of insurance (53.5% released vs 46.5% retained were uninsured; X2 =5.85, df =1; p<.016).

### Multivariate PES decision models

Since neither the quality of care nor the controls related to site and time of assessment significantly added to either of the two models, findings are presented for single stage logistic models including only those factors in our theoretical formulation. Six factors were significantly associated with unwanted release status among all patients in our first model (X2 = 109.01, df= 15; p< .0000, N = 451) (Table 2). Among psychiatric admission criteria, a three-point increase in one’s dangerousness score, a clinically significant elevation, was associated with a 180% decreased likelihood of being among those released yet wanting hospitalization when compared to all other patients. Not having a psychotic diagnosis was associated with a 17% higher likelihood of unwanted release. The most important factor associated with unwanted release, however, was availability of a less restrictive alternative to hospitalization. The availability of such an alternative is associated with a 2,414% greater likelihood of unwanted release compared to all other patients.

Those individuals with GAS scores that were five points higher than all others (the difference between the sample’s median of 35 and a score below 40 that is considered appropriate for acute treatment) had a 530% greater likelihood of being in the unwanted release group than other patients. Among the institutional constraints, being evaluated under difficult circumstances and the absence of insurance were respectively associated with a 395% and a 239% increased likelihood of being in the unwanted release group when contrasted with other patients. In considering only those patients who were seeking hospitalization, our second model in Table 2, indicates that only the admission criteria (i.e., lower dangerousness scores, a non-psychotic diagnosis, and the presence of a less restrictive alternative to hospitalization) as well as higher functional status were associated a greater likelihood of unwanted release (X2 = 84.754, df= 4; p< .0000; N = 451). This model allowed for a 91.67 % correct classification with only four false positives and four false negatives among 96 patients (one false positive patient’s status was attributable to a penal code commitment).

### Outcomes at 12 months

There were no deaths in the unwanted release group up to 12-months post evaluation as compared to 30(5.2%) among all other patients and 4 among those people who sought hospitalization and were retained. This finding approached statistical significance, with a X2 =2.67, df=1, p=.105 in the former comparison and X2 =2.63, df=1, p=.105 in the latter. Sixteen individuals in the unwanted release group were convicted of a crime within the eighteen months following their evaluation. No differences were found between this group and all other patients, or patients seeking hospitalization who were retained, in the rate of post-evaluation crime convictions. Eleven patients from the unwanted release group were admitted to the PES on an involuntary hold within a year following their evaluation. No differences were found between this group and other patients or patients seeking hospitalization who were retained in the rate of post-evaluation involuntary returns to the PES.

## Discussion

It would appear that denial of access to inpatient care is based upon the severity of the patient’s condition. PES clinicians seem to be adhering to a de facto form of rationing of inpatient care based upon the exhibition of such behavior that would make a patient admissible under California’s involuntary civil commitment standards. Clinicians appear to employ a strict conformity to these narrowly defined admission criteria in order to insure fairness in addressing need for inpatient hospitalization. Ideally, and in fact our results seem to indicate this, decisions for inpatient care allocation are made based upon the clinical assessment of dangerousness and the presence of a psychotic diagnosis when no less restrictive alternative to hospitalization is available. This was found to be true when considering the factors associated with membership in the unwanted release group as contrasted with all other patients, as well as when the contrast was made with those who were seeking hospitalization and were admitted. With respect to the unwanted release group, however, there also seems to be an effort to confirm the release decision against a level of functioning standard. This effort appears to result from the unusual circumstance of a patient preferring an inpatient disposition, even when a less restrictive alternative is available. Clinicians were likely to release only those patients preferring hospitalization who had a significantly higher GAS score-individuals averaging above 40, the acute treatment cutoff-and lower symptom severity scores.

Ironically, clinicians’ action with respect to the retention of those more dysfunctional individuals who meet the admission criteria and who are seeking hospitalization, is to confirm the patient’s status by admitting them as “involuntary” patients. This appears to take the ambiguity out of the situation, so that patients who later change their mind about wanting hospitalization do not then have to be involuntarily detained. Thus, if patients met involuntary admission standards they were involuntarily admitted, despite their willingness to be admitted voluntarily. This practice, however, usurps the patient’s agency in the decision to seek hospitalization, and may promote a sense of coercion in the experience of being hospitalized. The PES context is a difficult one in which to function both for the patient and the clinician. It becomes more difficult when the evaluation interview and efforts at collateral contacts are accompanied by relentless noise, limited space, limited phone access, visual distractions, and/or other negative stimuli. While the assessment of such conditions may be somewhat subjective, our observers all had at least a year of clinical work experience in a PES, and had visited more than one PES. Due to their work experience, observers were in a particularly good position to assess the presence of difficult circumstances during the patient evaluation.

Unwanted release, unfortunately, was more frequently accompanied by an assessment carried out under difficult circumstances. Though clinicians seem to have coped well with these difficult circumstances-adhering to the admission criteria and functioning standards in making their decisions the patient evaluated under such circumstances (and perhaps an accompanying family member), is probably likely to raise questions about the adequacy of the evaluation, as would any rational individual, let alone one desperate enough to seek inpatient psychiatric care. We need to pay further attention to the context of the assessment and its impact on the outcome of the PES evaluation. Those without insurance were more likely to be released, all other things being equal. While the hospital may not be the best choice for a treatment site when a less restrictive alternative is available, mistakes in denying access to care to this population on the basis of a lack of insurance can be very costly in terms of death and/or injury to both the individual and the community. The incentive to reduce hospitalization days is now a primary factor driving treatment decisions, and, as we have observed, is contributing to adverse consequences in the form of what appear to be premature releases and higher recidivism rates [25]. The data on the follow-up herein are not encouraging. Upon discharge patients face very limited community care resources, and often become homeless as a result. While no deaths were recorded in the unwanted release group, a quarter of those seeking hospitalization, regardless of their disposition, were involuntarily readmitted to the PES within 12 months, and 32% were convicted of a crime in this same period.

Involuntary outpatient commitment as a possible follow-up solution holds some potential [26,27]. The lack of investment in treatment resources is the biggest deficit. No matter how well the triage system operates, no matter how closely it conforms to the assessment criteria and deals with functional status as a part of insuring that individuals are not incorrectly released when they need care, failure to provide such care may lead to the occurrence of a Kendra Webdale event. While the findings of this study may not be replicable in other jurisdictions, they do represent the practice in nine different counties in California. Further, since no significant cross room effects were observed above and beyond those contributed by the model, we believe that the findings may be comfortably generalized to the rest of California, and, to a lesser extent, those other states using the dangerousness criterion as part of a crisis evaluation in the first phase of the civil commitment process. We also cannot know for sure that the 12 month outcomes are adverse in relation to the index PES evaluation. Much time has elapsed and, in the follow-up phase of the study we have had to rely on archival materials, not measuring other possible intervening factors to rule out their relative influence. Yet, to date this is the best outcome indication available with respect to a thorough empirical assessment of the PES process.

## Conclusion

Dangerousness and mental disorder in the absence of a less restrictive alternative to hospitalization, along with an overall assessment of the patient’s functional status, are effectively employed as triage criteria in determining who should be denied access to inpatient care following PES evaluation. While some higher functioning individuals are subjected to a variant standard of access to inpatient care because of a lack of insurance, and endure the misfortune of being evaluated under difficult clinical circumstances, outcomes seem contingent on the clinicians’ ability to distinguish between groups on the aforementioned triage criteria.

## Figures and Tables

**Table 1:** Symptom presentation on indicators of mental disorder for patients seeking admission by disposition.

Symptom	Disposition	N	Mean	SD	T *	P
Thought (form)	Released	18	1.94	1.39	−2.84	.006
	Retained	51	3.37	1.80		
Thought (content)	Released	18	2.11	1.53	−3.29	.002
	Retained	51	3.65	1.75		
Perception	Released	18	1.72	1.23	−2.74	.008
	Retained	51	2.80	1.92		
Orientation	Released	18	1.44	1.04	2.11	.039
	Retained	51	2.16	1.64		
Memory	Released	18	1.22	.94	−1.51	NS
	Retained	51	1.65	1.18		
Judgement	Released	18	3.22	1.66	2.34	.036
	Retained	51	4.20	1.46		
Behavior	Released	18	2.28	1.67	4.99	.000
	Retained	51	4.33	1.44		
Depression	Released	18	2.72	1.36	0.22	NS
	Retained	51	2.82	1.72		
Anxiety	Released	18	2.72	1.64	0.92	NS
	Retained	51	3.14	1.64		
Irritability	Released	18	1.89	1.23	3.18	.002
	Retained	51	3.24	1.63		
Expansiveness	Released	18	1.56	.92	1.65	NS
	Retained	51	2.06	1.52		
Impulsivity	Released	18	3.72	1.36	2.16	.03
	Retained	51	4.47	1.22		
Inappropriate Affect	Released	18	1.78	1.11	−1.42	NS
	Retained	51	2.31	1.45		

* t test is based on assumption of equal variances unless the group variances were found to be significantly different. In that case the data reported assume unequal variances.

**Table 2:** Logistic regressions of denial of inpatient care on predictive factors.

Dependent Variable	Denial of Inpatient Care					
Sample	All Patients (N=480)*			Patients Seeking Admission (N=96)*		
Method	All variables entered			Stepwise		
Statistics	b	p	Odds Ratio	b	p	Odds Ratio
**Psychiatric admission criteria:**						
Dangerousness	−.40	.000	.69	− .78	.000	.46
Less Restrictive Alternative Avail	3.18	.002	24.14	4.1	.002	60.51
Psychotic Disorder	−.83	.052	.43	− 1.84	.028	.16
Benefit from Hospital Stay	.42	NS**	NS	NE***	NE	NE
Treatability	−.57	NS	NS	NE	NE	NE
**Functional Status**						
GAS	.06	.010	1.06	.09	.03	1.09
**Institutional Constraints**						
Difficult Circumstances	1.37	.007	3.95	NE	NE	NE
No Insurance	.87	.045	2.39	NE	NE	NE
Workload	−.11	NS	NS	NE	NE	NE
**Procedural Justice Indicators**						
Advocate for Hospitalization	−.04	NS	NS	NE	NE	NE
Advocate Against Hospitalization	− .21	NS	NS	NE	NE	NE
Involuntary Entry to P.E.S.	.29	NS	NS	NE	NE	NE
Art of Care	1.14	NS	NS	NE	NE	NE
**Social Bias Indicators**						
Female Gender	−.22	NS	NS	NE	NE	NE
Client Ethnicity	.86	NS	NS	NE	NE	NE
*Model Statistics	Model X^2^ = 109.01, 15 df; p<.000			Model X^2^ = 84.75, 4df; p<.000		

** NS= Not Significant

*** NE=Not entered into equation because Log Likelihood decreased by less than .01 percent in stepwise entry.

## Abbreviations:

PES: Psychiatric Emergency Service
TRIAD: Three Ratings of Inpatient Admissibility
GAS: Global Assessment Scale
IMDS: Indicators of Mental Disorder Scale
